# Surface coatings of ZnO nanoparticles mitigate differentially a host of transcriptional, protein and signalling responses in primary human olfactory cells

**DOI:** 10.1186/1743-8977-10-54

**Published:** 2013-10-21

**Authors:** Megan J Osmond-McLeod, Ronald IW Osmond, Yalchin Oytam, Maxine J McCall, Bryce Feltis, Alan Mackay-Sim, Stephen A Wood, Anthony L Cook

**Affiliations:** 1 CSIRO Advanced Materials TCP (Nanosafety), and CSIRO Animal, Food and Health Sciences, PO Box 52, North Ryde, NSW 1670, Australia; 2 TGR Biosciences, Thebarton, SA 5031, Australia; 3 School of Medical Sciences, RMIT University, Bundoora, VIC 3083, Australia; 4 Centre for Green Chemistry, Monash University, Clayton, VIC 3800, Australia; 5 National Centre for Adult Stem Cell Research, Eskitis Institute for Cell and Molecular Therapies, Griffith University, Brisbane, QLD 4111, Australia; 6 Current address: School of Human Life Sciences, University of Tasmania, Launceston, TAS 7250, Australia

**Keywords:** Zinc oxide, Nanoparticle, Olfactory, Gene expression, Cell-signalling, DNA-damage

## Abstract

**Background:**

Inhaled nanoparticles have been reported in some instances to translocate from the nostril to the olfactory bulb in exposed rats. In close proximity to the olfactory bulb is the olfactory mucosa, within which resides a niche of multipotent cells. Cells isolated from this area may provide a relevant *in vitro* system to investigate potential effects of workplace exposure to inhaled zinc oxide nanoparticles.

**Methods:**

Four types of commercially-available zinc oxide (ZnO) nanoparticles, two coated and two uncoated, were examined for their effects on primary human cells cultured from the olfactory mucosa. Human olfactory neurosphere-derived (hONS) cells from healthy adult donors were analyzed for modulation of cytokine levels, activation of intracellular signalling pathways, changes in gene-expression patterns across the whole genome, and compromised cellular function over a 24 h period following exposure to the nanoparticles suspended in cell culture medium.

**Results:**

ZnO nanoparticle toxicity in hONS cells was mediated through a battery of mechanisms largely related to cell stress, inflammatory response and apoptosis, but not activation of mechanisms that repair damaged DNA. Surface coatings on the ZnO nanoparticles mitigated these cellular responses to varying degrees.

**Conclusions:**

The results indicate that care should be taken in the workplace to minimize generation of, and exposure to, aerosols of uncoated ZnO nanoparticles, given the adverse responses reported here using multipotent cells derived from the olfactory mucosa.

## Background

Zinc oxide (ZnO) nanoparticles have remarkable ultraviolet (UV) absorbing, optical and optoelectronic properties that make them valuable for a variety of commercial applications [1], including use in sunscreen products where their transparency on the skin and the protection they provide against broad-spectrum UV radiation [2,3] is of consumer benefit. However, with increasing commercial application comes the potential for increased workplace exposure to airborne particles (reviewed in [4]). Inhalation of ZnO fumes - which can include particles in the nanometre range - is associated with the onset of metal fume fever, an illness characterized by transitory pulmonary and systemic alterations in humans [5]. Recent *in vivo* studies have reported the onset of oxidative stress, inflammation, and lung injury following intratracheal instillation or inhalation of ZnO nanoparticles in rats [6-9]. Numerous *in vitro* experiments have also pointed to cell injury caused by ZnO nanoparticles, or Zn^2+^ from partially dissolved particles (e.g. [10-14]). However, there are no known long-term effects of ZnO fume inhalation, and there is some evidence that, whilst initial exposures can induce a pulmonary inflammatory response [15-17], humans may develop tolerance to inhaled ZnO fumes upon repeated exposure [18].

Surface coatings are added to ZnO nanoparticles for ease of handling and to modulate their properties. For example, coating facilitates their dispersability in the oil phase of sunscreen formulations, as well as improving the texture of the sunscreens on skin [19]. From a nanotoxicological perspective, stable surface coatings have been reported to suppress the generation of reactive oxygen species (ROS) by ZnO nanoparticles [20,21] and may also decrease the propensity for ZnO nanoparticles to dissolve in biological environments. Thus, surface coating may mitigate two postulated mechanisms of ZnO nanoparticle-mediated cytotoxicity.

Following inhalation by rats, some types of nanoparticles (graphite nanorods, manganese oxide and gold) have been shown to accumulate in the olfactory bulb after depositing on the olfactory mucosa and translocating along the olfactory neuronal pathway [22-24]. This has led to interest in the effects of nanoparticles on neural cells and brain function [13,25,26], as well as the potential application of this pathway for drug delivery systems [27]. Within the olfactory mucosa reside a niche of cells that, when cultured *in vitro*, can form neurospheres that contain multipotent cells that can differentiate to neurons and glial cells [28-30]. Given the multipotent nature of this cell population, as well as its proximity to a site of deposition of nanoparticles following nasal inhalation, their response to nanoparticle exposure is relevant in assessing the potential for adverse effects following possible workplace exposure to airborne nanoparticles.

Here, we have assayed the response of human olfactory neurosphere-derived (hONS) cells established from adult donors [31] to ZnO nanoparticles. To assess the potential for altered cellular responses mediated by different types of surface coatings, we tested two coated and two uncoated (but different sized) ZnO nanoparticles. The ZnO nanoparticles selected for study are all manufactured in large scale, available commercially, and used in commercially-available products. For a thorough assessment of the biological effects of these ZnO nanoparticles, we employed a systems approach, assaying a wide range of cellular responses – cytokine release, cell-signalling, whole-genome transcriptional profiling, and cell viability, stress and metabolism – to determine whether early responses to ZnO exposure are reflected by changes in cellular function.

We found that cells treated with the ZnO nanoparticles showed generally robust and internally consistent responses across a wide range of biological endpoints, with uncoated nanoparticles eliciting greater cellular stress and cytotoxicity compared to coated ZnO nanoparticles. Further, the surface coatings served to either delay, or largely mitigate, the adverse cellular responses, depending on the composition, and possibly other characteristics, of the coating.

## Results

**Figure 1:**
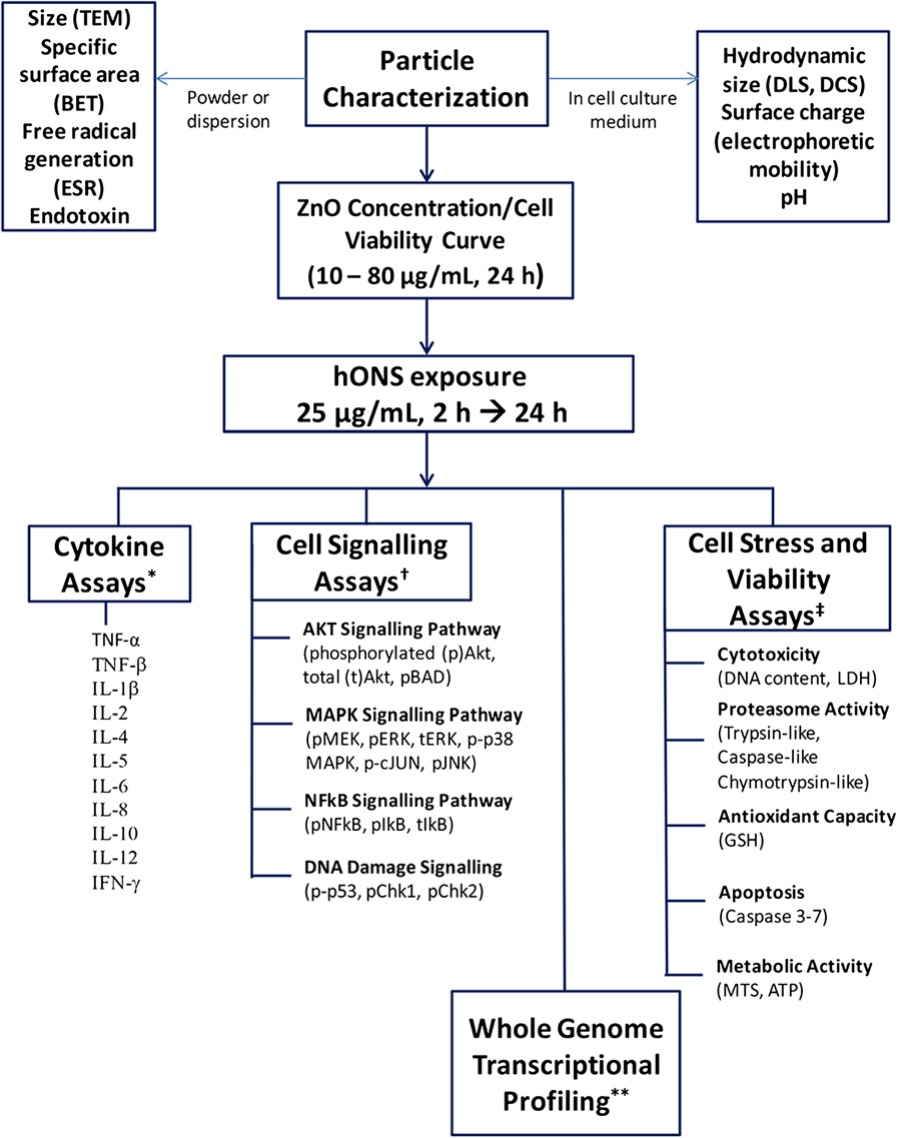
**Experimental overview.** Nanoparticles were characterised as powders or dispersions in aqueous media (top left), and also as dispersions in DMEM cell-culture medium (top right). An initial concentration response curve was generated to select an appropriate treatment concentration that elicited a mechanistic response in hONS cells for at least one of the ZnO products. hONS cells were exposed to ZnO products at the selected concentration (25 μg/mL) for up to 24 h, after which the cellular responses to treatment were measured by a variety of assays. ^‡^ Cells from four human donors, A, B, C and D, each in three replicate wells for each treatment time-point (2 h, 6 h and 24 h); ^*^ Donor A cells, in three replicate wells for each treatment time-point (2 h and 6 h); ^†^ Donor A cells, in four replicate wells for each treatment time-point (2 h, 4 h, 6 h, 8 h, and 10 h); ^**^ Donor A cells, in four replicate wells for each treatment time-point (2 h and 6 h).

A scheme summarising our experimental approach, including particle characterisation and specific assays used to measure cellular responses to the nanoparticles, is shown in Figure 1. Four types of commercially-available ZnO particles were assessed. Two were uncoated (Z-COTE from BASF, and Nanosun P99/30 (hereafter referred to as Nanosun) from Micronisers) and two were coated (Z-COTE HP1 and Z-COTE MAX, both from BASF). Z-COTE HP1 (referred to as HP1) is coated with triethoxycaprylylsilane, and Z-COTE MAX (referred to as MAX) is coated with a dimethoxydiphenylsilane/triethoxycaprylylsilane crosspolymer. Our first experiments were nine cell stress and viability assays (lower-right side of Figure 1), using hONS cells derived from each of four human donors A, B, C and D. Three replicate wells for each ZnO treatment time-point were used for each assay. While some biological variation was present between the four donors, treatment effects typically far outweighed biological variations between the donors. For subsequent experiments (cytokine, cell signalling, and whole-genome gene expression) we used cells from donor A, where three to four replicate wells for each treatment time-point were used. Thus, by generating information on a very large number and variety of endpoints, the systemic responses of hONS cells exposed to commercial samples of coated and uncoated ZnO nanoparticles could be identified.

### Nanoparticle characterisation

The properties of particles can alter in a size-dependent manner, and concomitant differences in induced cytotoxicity have been variously ascribed to physicochemical characteristics including particle size, surface area, shape, surface charge and free radical generation [32]. Therefore, we measured several physicochemical properties of the nanoparticles used for the cellular experiments, as summarised in Table 1.

**Table 1:** Physical and chemical properties of the four types of ZnO nanoparticles

		**Z-COTE**	**Nanosun**	**HP1**	**MAX**	**Media/H**_**2**_**O only**
**Powder**	**Coating**	None	None	Triethoxycaprylylsilane	Dimethoxydiphenylsilane/Triethoxycaprylylsilane crosspolymer	N/A
	**Batch number**	EHDA3001	4051	CNHE0602	FCHE1301	N/A
	**TEM mean primary size (nm)**	**Width**^**+**^**:** 44 ± 12	**Diameter:** 25 ± 1	**Width:** 28 ± 2	**Width:** 36 ± 2	N/A
		**Length:** 73 ± 3		**Length:** 96 ± 6	**Length:** 95 ± 6	
	**BET total surface area (m**^**2**^**/g) (#1)**	14.5	32.5	14.4	10.8	N/A
	**BET total surface area (m**^**2**^**/g) (#2)**	12.3	28.2	15.4	12.7	
**In H**_**2**_**O**	**Generation of peroxynitrile or**	**Dark:** 800 ± 300*	**Dark:** 280 ± 80	**Dark:** 220 ± 40	**Dark:** 170 ± 50	N/A
	**superoxide radicals (% negative control)**	**Light:** 12,000 ± 3000*	**Light:** 2000 ± 400	**Light:** 4000 ± 700	**Light:** 4,000 ± 300	
	**Endotoxin**	BD	BD	BD	BD	N/A
	**DLS average particle size (nm) (PDI in parenthesis)**	**0-time:** 410 (0.34)	**0-time:** 600 (0.56)	**0-time:** 240 (0.25)	**0-time:** broad (0.96)	**0-time:** N/A
	**Zeta potential (mV)**	**0-time:** 27	**0-time:** 9	**0-time:** -10	**0-time:** -7	**0-time:** -3
	**Altered pH (relative to H**_**2**_**O only)**	**0-time:** -0.1	**0-time:** 0.0	**0-time:** -0.1	**0-time:** -0.1	**0-time:** N/A
**In cell culture medium (DMEM:F12, 1:1)**	**DLS average particle size (nm) over**	**0-time:** 500 (0.54)	**0-time:** 240 (0.31)	**0-time:** 360 (0.36)	**0-time:** 150 (0.21)	**0-time:** 20 (0.39)
	**24 h (PDI in parenthesis)**	**2 h:** 300 (0.47)	**2 h:** 350 (0.36)	**2 h:** 120 (0.17)	**2 h:** 150 (0.21)	**2 h:** 20 (0.42)
		**6 h:** 400 (0.49)	**6 h:** 600 (0.57)	**6 h:** 100 (0.15)	**6 h:** 150 (0.21)	**6 h:** 20 (0.40)
		**24 h:** 360 (0.36)	**24 h:** 440 (0.44)	**24 h:** 260 (0.28)	**24 h:** 203 (0.25)	**24 h:** 10 (0.39)
	**DCS particle size distribution (nm)**	**0-time:** 100-900+	**0-time:** BD	**0-time:** 150-900+	**0-time:** 100-400	**0-time:** BD
	**Zeta potential over 24 h (mV)**	**0-time:** -9	**0-time:** -8	**0-time:** -10	**0-time:** -7	**0-time:** -6
		**2 h:** -8	**2 h:** -8	**2 h:** -8	**2 h:** -4	**2 h:** -6
		**6 h:** -10	**6 h:** -8	**6 h:** -5	**6 h:** -6	**6 h:** -6
		**24 h:** -13	**24 h:** -9	**24 h:** -6	**24 h:** -8	**24 h:** -6
	**Altered pH of media over 24 h (relative to media only)**	**0-time:** +0.1	**0-time:** -0.1	**0-time:** +0.1	**0-time:** +0.2	N/A
		**2 h:** +0.2	**2 h:** 0.0	**2 h:** +0.1	**2 h:** -0.2	
		**6 h:** -0.1	**6 h:** +0.1	**6 h:** -0.1	**6 h:** 0.0	
		**24 h:** -0.2	**24 h:** 0.0	**24 h:** 0.0	**24 h:** 0.0	

Nanoparticles were characterised as dry powders or as dispersions in either water or cell-culture medium. For measurements of hydrodynamic diameters of particles or agglomerates by DLS, polydispersity indices (PDI) are given in parentheses. N/A not applicable; NS not significant; BD below detection; * statistically significant compared to negative control (saline only); ^+^ errors are standard errors of means.

### Particle size and shape

**Figure 2:**
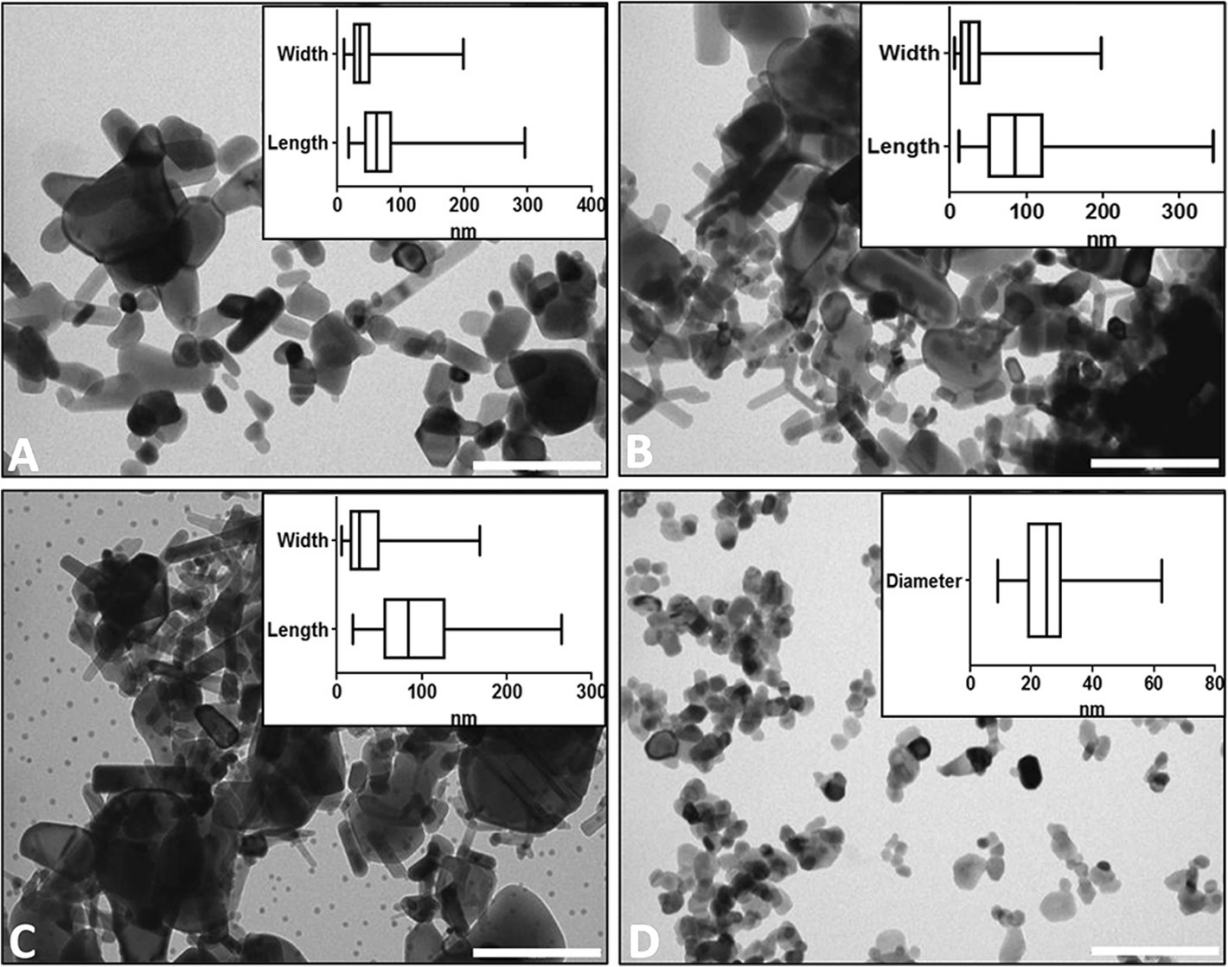
**Nanoparticle morphologies and size distributions.** TEM images of ZnO nanoparticles used in this study alongside boxplots showing the distributions of particle lengths and widths (**A**. Z-COTE; **B**. HP1; **C**. MAX) or diameter (**D**. Nanosun), depending on whether particles were mainly rod-shaped or spherical, respectively. 90–300 measurements were made for each dimension. The vertical line in the box represents the median value and the edges of the box represent the lower and upper quartiles. The whiskers at the ends of the horizontal lines represent minimum and maximum values. Scale bar for TEM images = 200 nm.

The three BASF products, Z-COTE, HP1 and MAX, are typically rod-shaped, with heterogeneous sizes ranging up to ~350 nm, as assessed by transmission electron microscopy (TEM). In general, the uncoated Z-COTE particles are wider (average of 44 nm) and shorter (73 nm) than the coated HP1 (28 nm, 96 nm) and coated MAX (36 nm, 95 nm) (Table 1). These sizes are broadly consistent with the manufacturer’s specifications, which describe particle size as <200 nm. In contrast, the uncoated Nanosun sample, from Micronisers, consists of mostly spheroidal particles, and shows a comparatively tighter size distribution with an average particle diameter of 25 nm, consistent with the manufacturer’s specifications (30 nm). The morphology for each type of nanoparticle in its powder form can be seen in TEM images, with their mean sizes and size distributions indicated in box-plots, in Figure 2.

### Specific surface area

From nitrogen-adsorption isotherms determined for each ZnO sample in powder form, specific surface areas of the ZnO samples were calculated using the Brunauer-Emmett-Teller (BET) equation. Two sets of experiments were performed independently by different operators using different equipment; there were no substantial differences between the two data sets. Consistent with its small size, Nanosun had the largest surface area (30±3 m^2^/g, average of the two measurements in Table 1), whereas Z-COTE and HP1 were similar (13±2 and 14.9±0.5 m^2^/g, respectively), and also very similar to MAX (12±1 m^2^/g) which had the smallest surface area; the last three values are at the lower end of the manufacturer’s specifications of 12–24 m^2^/g.

### Surface coatings on HP1 and MAX

Analysis of elements in the two coated ZnO samples, by ICP-AES (inductively coupled plasma atomic emission spectroscopy), revealed a larger percentage weight of silicon in MAX (0.21%) compared with HP1 (0.17%), indicating a greater number of silicon-containing, surface-coating molecules in MAX than in HP1. Thermogravimetric analyses (TGA) of the same samples, whereby changes in weight upon heating samples to high temperatures are very accurately measured, revealed total percentage weight losses that were greater for MAX (2.15%) compared with HP1 (1.74%), consistent with the thermal decomposition of more surface molecules in MAX than HP1. Given that the coating on MAX is a dimethoxydiphenylsilane-triethoxycaprylylsilane crosspolymer while that on HP1 is pure triethoxycaprylylsilane, the molecular weight of dimethoxydiphenylsilane is less than triethoxycaprylylsilane, and the specific surface area of MAX is slightly smaller than that of HP1, the ICP-AES and TGA data reveal that MAX has more surface-coating molecules per unit surface area than does HP1. This could be manifested by MAX having a thicker coating than HP1, or a greater extent of surface coverage, or both.

### Free radical generation

The nanoparticles dispersed in saline solution were assessed by electron paramagnetic resonance (EPR) for their ability to generate peroxynitrile or superoxide radicals using TEMPONE-H as a spin trap. All samples generated these radicals above control levels, and all samples generated more radicals in the presence of light than when incubated in darkness. However, only Z-COTE was found to generate statistically significant levels compared to the negative control in both the light and the dark (Table 1). In addition, Z-COTE generated levels of peroxynitrile and superoxide radicals significantly higher than MAX, but not HP1 or Nanosun, in the dark, and significantly higher than HP1, MAX, and Nanosun in the light.

### Detection of endotoxins on nanoparticles

Levels of endotoxin in all four ZnO products were below detection limits (Table 1).

### Characterisation of nanoparticles dispersed in water and cell culture medium

When hydrodynamic size in water or DMEM cell-culture medium was measured, the primary particle was rarely seen, and, instead, particle agglomerates were detected as measured by both dynamic light scattering (DLS) or differential centrifugation sedimentation (DCS) for all particle types (Table 1). Of the four particle types, MAX formed the largest agglomerates in water, and the smallest in cell culture medium, when initially dispersed. With increasing time, both the coated particles, HP1 and MAX, formed smaller agglomerates in medium compared with both types of uncoated ZnO nanoparticles.

Both uncoated nanoparticles, Z-COTE and Nanosun, had positive surface charges in water, while both coated nanoparticles, HP1 and MAX, appeared to have negative surface charges, as indicated by their zeta potentials (Table 1). However, negative surface charges were observed for all four products in cell culture medium, and these were generally close to that observed for the medium itself, suggestive of medium characteristics dominating over innate particle surface charge.

The pH of solutions containing nanoparticles was typically found to be within −0.1 of nanoparticle-free water, and within ±0.2 of cell culture medium over the 24 h incubation under cell culture conditions, suggesting that the minimal impact the nanoparticles may have had on pH was effectively buffered by the medium (Table 1).

### Selection of nanoparticle mass concentration used for experiments with cells

All results described below are from experiments with cells treated with an applied mass-equivalent concentration of 25 μg/mL for each of the four different ZnO samples, and compared with medium-only controls. This concentration of 25 μg/mL was based on preliminary experiments that showed measureable cellular cytotoxicity within the experimental time-frame used here (Additional file 1: Figure S1).

### Cytokine assays

**Figure 3:**
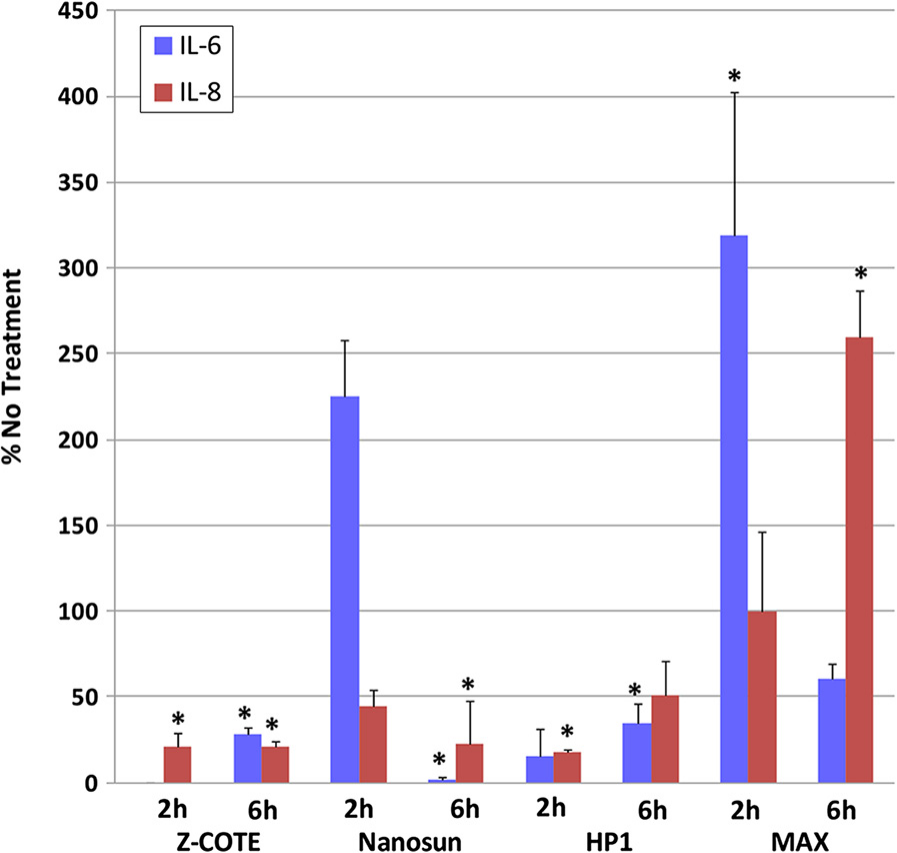
**Levels of the cytokines IL-6 and IL-8 in hONS cells treated with ZnO nanoparticles.** hONS cells from donor A were placed in three replicate wells for each ZnO treatment time-point (2 h and 6 h). Results were averaged and expressed relative to levels in time-matched untreated cells set as 100%. * Indicates statistical significance relative to untreated cells.

Figure 3 shows levels of the pro-inflammatory cytokines IL-6 and IL-8 secreted from hONS cells from donor A after exposure to ZnO particles for 2 h and 6 h, relative to levels in cells not exposed to nanoparticles, taken as 100%, for each time point. IL-6 and IL-8 were secreted at levels significantly above those in untreated cells at 2 h and 6 h, respectively, in cells exposed to the coated nanoparticle, MAX. IL-6 was also elevated in Nanosun-treated cells at 2 h, but not in cells treated with Z-COTE or HP1, where levels were not detected or were strongly suppressed, respectively. By 6 h exposure, the levels of IL-6 had dropped sharply in cells treated with Nanosun and MAX to below those detected in untreated cells, and levels in cells treated with Z-COTE and HP1 were also well below those in untreated cells. Relative to no treatment, IL-8 was strongly suppressed at both 2 h and 6 h exposure for all treatments except MAX. TNF-α, TFN-β, IL-1β, IL-2, IL-4, IL-5, IL-10, IL-12 and IFN-γ were also assayed, but levels were below the detection limit (20 pg/mL) for all treatments, consistent with previous work showing that the secretion of most of these cytokines was not stimulated by ZnO nanoparticles [33].

### Cell-signalling assays

**Figure 4:**
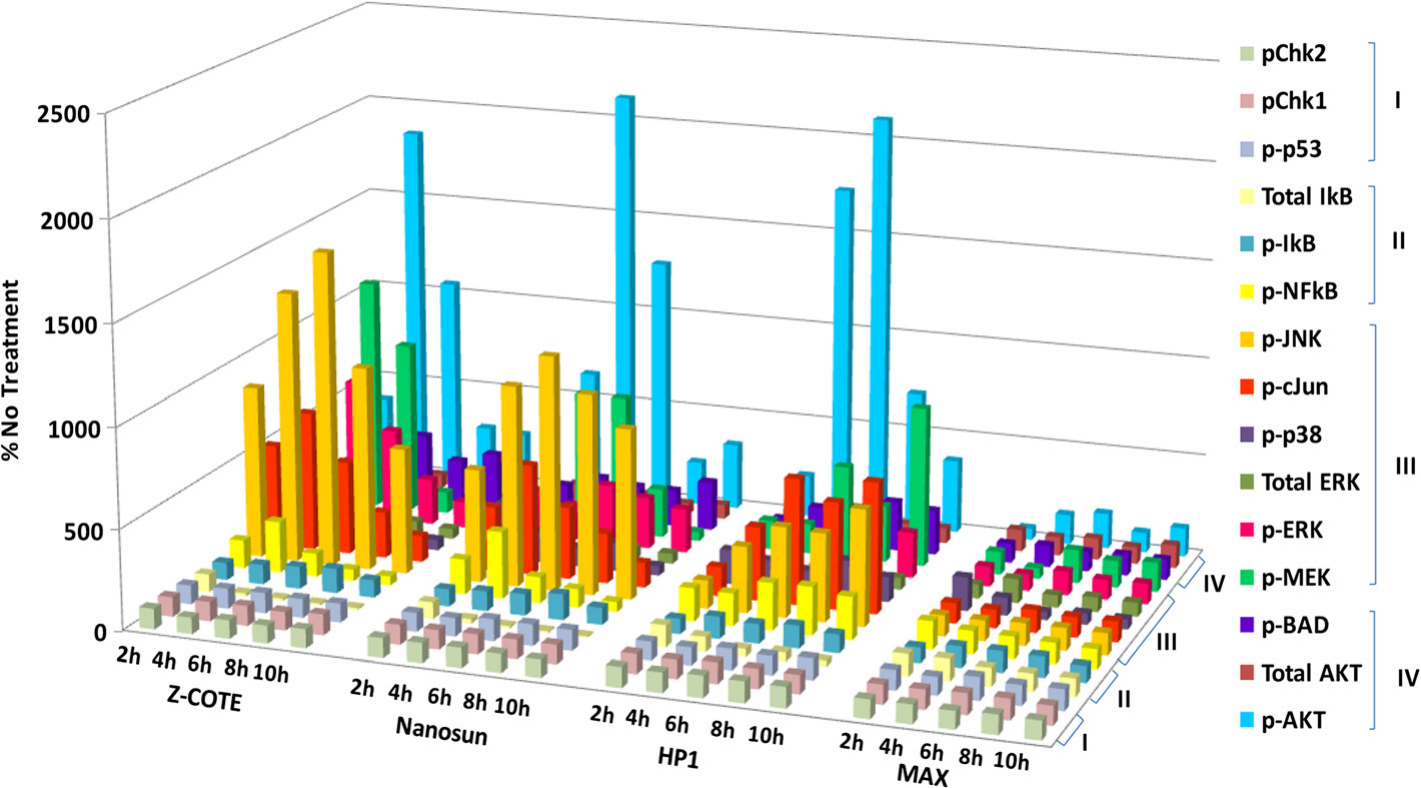
**Phosphorylation of key proteins from four major cell-signalling pathways in hONS cells treated with ZnO nanoparticles.** hONS cells from donor A were placed in four replicate wells for each ZnO treatment time-point (2, 4, 6, 8 and 10 h). Levels of phosphorylation of proteins involved in the selected cell-signalling pathways in cell lysates were averaged and expressed as the percentage of time-matched untreated cells set as 100%. The four cell-signalling pathways are I DNA Damage; II NFκB; III MAPK; IV AKT. Data used to generate this figure, and associated standard errors of means and statistical significance, are supplied separately in Additional file 2: Table S1.

hONS cells from donor A were tested for nanoparticle-mediated activation of several cell-signalling pathways by assaying the levels of phosphorylation (p) of key proteins involved in those pathways (Figure 1). Activation of MAPK/ERK pathways was assessed by measuring the levels of pMEK and pERK (cell survival, growth and differentiation), and pJNK, p-cJUN and p-p38 (inflammation, apoptosis, growth and differentiation). Activation of the AKT signalling pathway, associated with cell apoptosis and survival, was assessed by measuring the levels of pAkt and pBAD. The NF-κB pathway, associated with cellular stress response, was assessed by measuring the levels of pNF-κB and pI-κB. Perturbations of DNA-damage response pathways were assessed by the levels of pChk1, pChk2 and p-p53. Total (t) ERK, tAkt and tI-κB levels were also monitored in the cellular lysates, to ensure that the concentration of the signalling proteins was not changing throughout the time course of the experiments. Measurements were made in cells after 2, 4, 6, 8 and 10 h exposure. Results from all cell-signalling experiments across all time-points are summarised in Figure 4. As this 3D form of presentation cannot include error bars, the data used to generate this figure, together with standard errors of means and statistical significance, are supplied separately in Additional file 2: Table S1.

Cells treated with the uncoated nanoparticles, Z-COTE and Nanosun, or the coated nanoparticle, HP1, showed similar cell-signalling profiles. Peaks of activity for both uncoated nanoparticles were generally observed by 6 h exposure, with signalling spikes diminishing thereafter, possibly due to treatment-mediated cell death at later time-points (see below). Peaks of activity for HP1-treated cells often occurred slightly later than for cells treated with either of the uncoated nanoparticles (Z-COTE, Nanosun), suggesting a slightly delayed response to the coated HP1 nanoparticles. The cell survival, growth and differentiation (pMEK, pERK) and inflammatory/apoptosis arms (pJNK, p-cJUN, p-p38) of the MAPK/ERK signalling pathway were the most strongly activated by these treatments (shown as pathway III in Figure 4). The Akt (pAkt, pBAD) and NF-κB (pNF-κB, pI-κB) signalling pathways were also significantly activated (pathways IV and II, respectively, in Figure 4), signifying that anti-apoptotic and inflammatory pathways in addition to MAPK had been induced. These cell stress/survival signalling pathways were also activated in cells treated with the coated MAX, but to a much smaller extent, rarely reaching statistical significance. The signalling pathway for response to DNA damage (pathway I, Figure 4) was not activated by any ZnO treatment.

### Analysis of gene expression from microarray data

hONS cells from donor A were exposed to the four types of ZnO particles for 2 h and 6 h, and levels of RNA transcripts representing 28,869 genes were measured using Affymetrix GeneChip Human Gene 1.0ST microarrays. Microarray data are freely available on the GEO Archive (http://www.ncbi.nlm.nih.gov/geo/) under accession number GSE45322.

**Figure 5:**
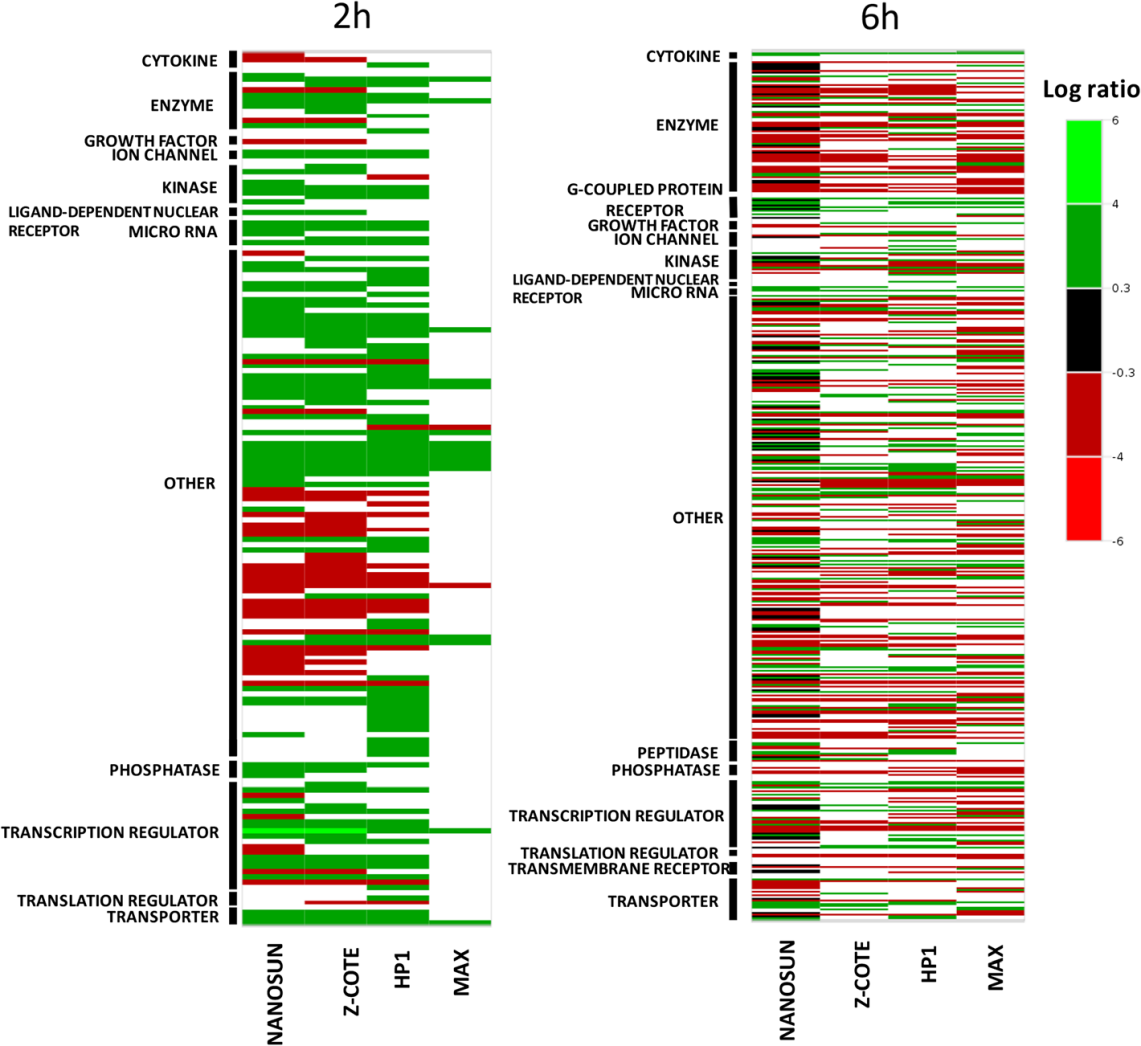
**Map of differential transcript activity in hONS cells treated with ZnO nanoparticles.** hONS cells from donor A were assessed in quadruplicate wells for each ZnO treatment time-point (2 h and 6 h). Differentially-expressed transcripts were grouped according to function, ordered alphabetically within each function, and arrayed alongside one another for each of the treatment data sets. The sorted files were then uploaded to the web-based prettygraph (http://www.prettygraph.com) to generate colour-coded maps for the aligned transcriptional profiles across all treatments. Green indicates up-regulated transcripts, red indicates down-regulated transcripts, and black signifies little difference from untreated cells (although still reaching statistical significance where *p*<0.05). White gaps signify treatments where transcripts were not differentially-expressed relative to untreated cells. The bar at the right hand-side shows the colour-code for the magnitude of the log ratio.

The Canonical Pathways most significantly activated in ZnO-treated cells compared to untreated cells were identified using Ingenuity Pathway Analysis (IPA) software. A list of the five Canonical Pathways most perturbed by exposure to each ZnO sample at 2 h and 6 h is shown in Table 2, and maps of differential transcript activity are given in Figure 5. In general, short exposures (2 h) to ZnO nanoparticles activated pathways involved in cellular stress responses, whereas longer exposures (6 h) perturbed pathways more related to cell injury and repair. All ZnO products activated similar pathways at 2 h, and particularly up-regulation of the “Aldosterone Signalling in Epithelial Cells Pathway” and the “NRF2-Mediated Oxidative Stress Response Pathway” (Table 2). The Aldosterone Signalling Pathway is implicated in the activity of transport proteins, or possibly cellular differentiation to modify electrolyte transport [34], and the NRF2-Mediated Oxidative Stress Response Pathway is associated with a cell survival response in the face of environmental toxicants [35]. By 6 h, however, a generalised down-regulation of a diverse range of Canonical Pathways was observed, suggestive of an overall decrease in transcription of specific genes potentially involved in these pathways with longer exposures, although the overall number of transcripts differentially regulated at 6 h was greater than at 2 h.

**Table 2:** Canonical pathways most significantly perturbed at the transcriptional level in hONS cells exposed to ZnO nanoparticles

**Particle**	**Top 5 Canonical pathways perturbed at 2 h**	***p - *** **value**	**Top 5 Canonical pathways perturbed at 6 h**	***p *** **value 2**
**Z-COTE** uncoated	Aldosterone signalling in epithelial cells	1.97E - 10	Nucleotide excision repair pathway	1.30E - 05
	NRF2-mediated oxidative stress response	1.33E - 08	Endoplasmic reticulum stress pathway	2.75E - 04
	Protein ubiquitination pathway	3.66E - 06	Protein ubiquitination pathway	6.82E - 04
	Glucocorticoid receptor signalling	3.53E - 05	Ubiquinone bisoynthesis	9.82E - 04
	Huntingtons’s disease signalling	7.91E - 05	Assembly of RNA Polymerase II complex	1.24E - 03
**Nanosun** uncoated	Aldosterone signalling in epithelial cells	4.70E - 07	Ubiquinone biosynthesis	9.92E - 12
	NRF2-mediated oxidative stress response	1.31E - 05	Mitochondrial dysfunction	4.00E - 11
	IL-17A signalling in fibroblasts	6.40E - 05	Nucleotide excision repair pathway	5.92E - 11
	Production of nitric oxide and reactive oxygen species in macrophages	1.17E - 04	Oxidative phosphorylation	2.70E - 09
	Glucocorticoid receptor signalling	1.87E - 04	Protein ubiquitination pathway	1.60E - 08
**HP1** coated (Triethoxycaprylysilane)	Aldosterone signalling in epithelial cells	4.12E - 07	Glucocorticoid receptor signalling	1.25E - 07
	NRF2-mediated oxidative stress response	1.17E - 06	Aldosterone signalling in epithelial cells	5.85E - 06
	Glucocorticoid receptor signalling	1.50E - 04	Protein ubiquitination pathway	8.79E - 06
	IL - 17A signalling in fibroblasts	4.88E - 04	NRF2-mediated oxidative stress response	7.27E - 05
	Protein ubiquitination pathway	9.24E - 04	Assembly of RNA Polymerase II complex	9.05E - 05
**MAX** coated (Dimethoxydiphenylsilane/triethoxycaprylylsilane cross-polymer)	Aldosterone signalling in epithelial cells	7.40E - 04	Hereditary breast cancer signalling	6.11E - 06
	NRF2-mediated oxidative stress response	1.01E - 03	Aminoacyl-tRNA biosynthesis	4.24E - 05
	IL-10 signalling	2.71E - 03	Glucocorticoid receptor signalling	5.61E - 05
	Protein ubiquitination pathway	2.85E - 03	Mismatch repair in eukaryotes	7.59E - 05
	Endothelin-1 signalling	1.48E - 02	Nucleotide excision repair pathway	1.04E - 04

Analysis at the transcript level revealed that the majority (over 50%) of transcriptional changes for all treatments was classified by the IPA system as “other”, and comprised gene products including molecular chaperones, metallothioneins, ribosomal proteins, and small nucleolar RNAs (Figure 5). The other broad functional categories that were substantially perturbed across all treatments comprised transcription regulators, enzymes, and transporters. Several more categories that were differentially regulated by treatment with Z-COTE, Nanosun or HP1 at 2 h, such as ion channels, kinases, microRNAs and phosphatases, were not perturbed in cells treated with MAX at the same time-point. By 6 h, however, both the range of categories and relative proportions of genes therein were essentially identical for all - although there were differences at the level of individual genes.

**Figure 6:**
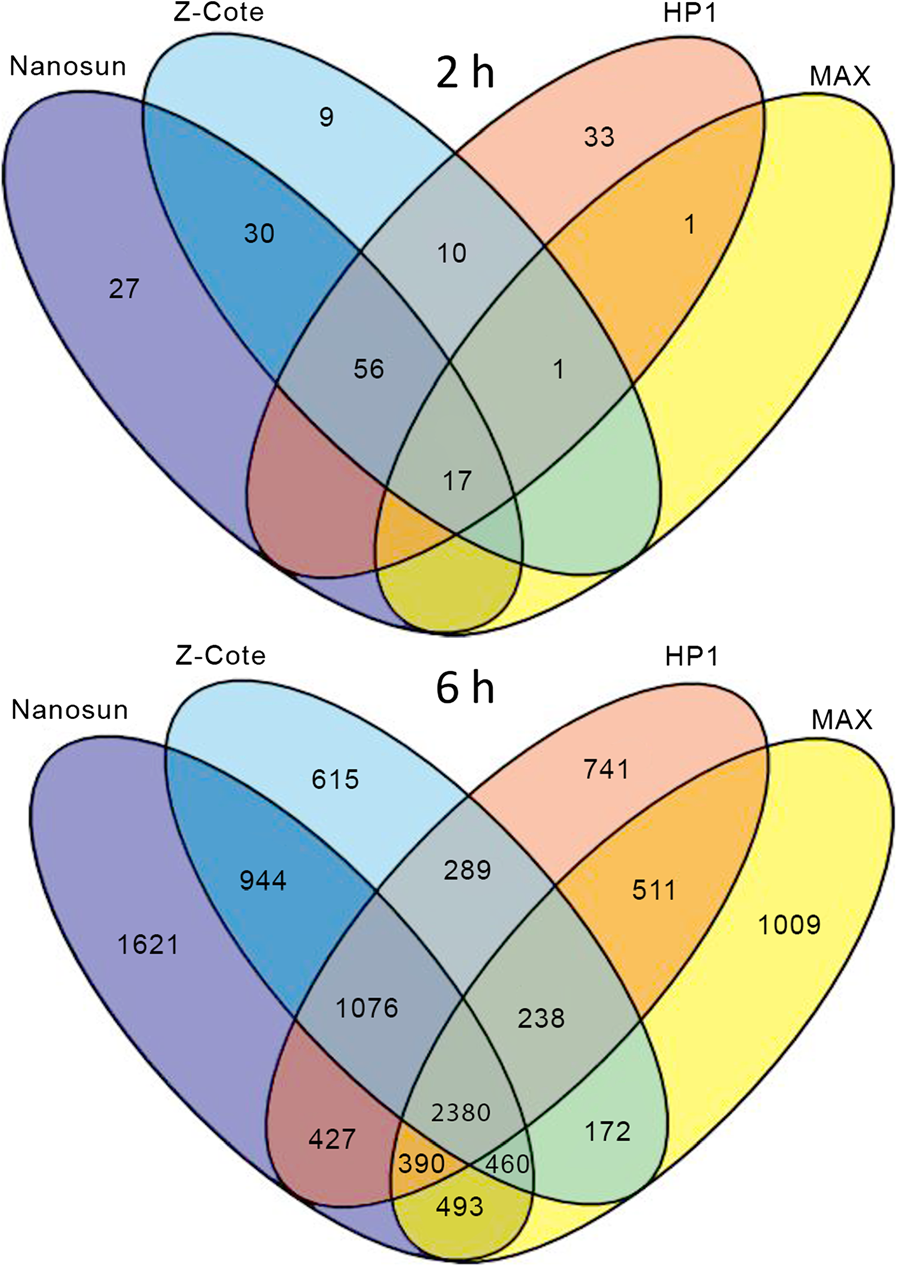
**VENN diagrame showing numbers of unique or shared transcripts within and between different ZnO treatments at 2 h and 6 h.** Numbers indicate differentially-expressed transcripts, graphed according to whether they occurred uniquely within one treatment (unshared VENN), or whether they were differentially activated by two or more of the treatments (intersecting VENN). Blank intersections indicate that no genes were unique to that intersection or treatment.

To gain a greater insight into the degree of transcriptional variations between treatments, the numbers of unique or shared transcripts across all treatments were counted and plotted in four-way VENN diagrams (Figure 6). At 2 h, the largest number of overlapping transcripts was shared between the uncoated particles (Z-COTE and Nanosun) and the coated HP1 (56 shared transcripts). Thirty transcripts were uniquely shared by Nanosun and Z-COTE, and 17 were shared by all treatments. However, the transcripts that had been uniquely activated by the cytotoxic treatments (Z-COTE, Nanosun, HP1) at 2 h, were also activated by MAX at 6 h, and thus were not ultimately unique to a particular ZnO product. In addition, when these reduced gene lists at 2 h were scrutinised, we found that they comprised transcripts already flagged in Canonical Pathways previously identified using the complete datasets, and no new pathways specific to these intersecting genes were highlighted. The total number of differentially-expressed genes was overall much greater at 6 h than 2 h and, as might be expected with such a large dataset, more genes populated each intersect of the VENN diagram. Nevertheless, by far the greatest number of genes that were differentially expressed was largely shared by all treatments (2380 transcripts). Furthermore, transcripts that were unique to individual treatments or specific intercepts once again, and for the same reasons as discussed above, did not convincingly translate to unique pathways being activated by these treatments when assessed by IPA, but simply appeared to contribute to a stronger perturbation of pathways that had already been identified.

### Assays for cellular function

**Figure 7:**
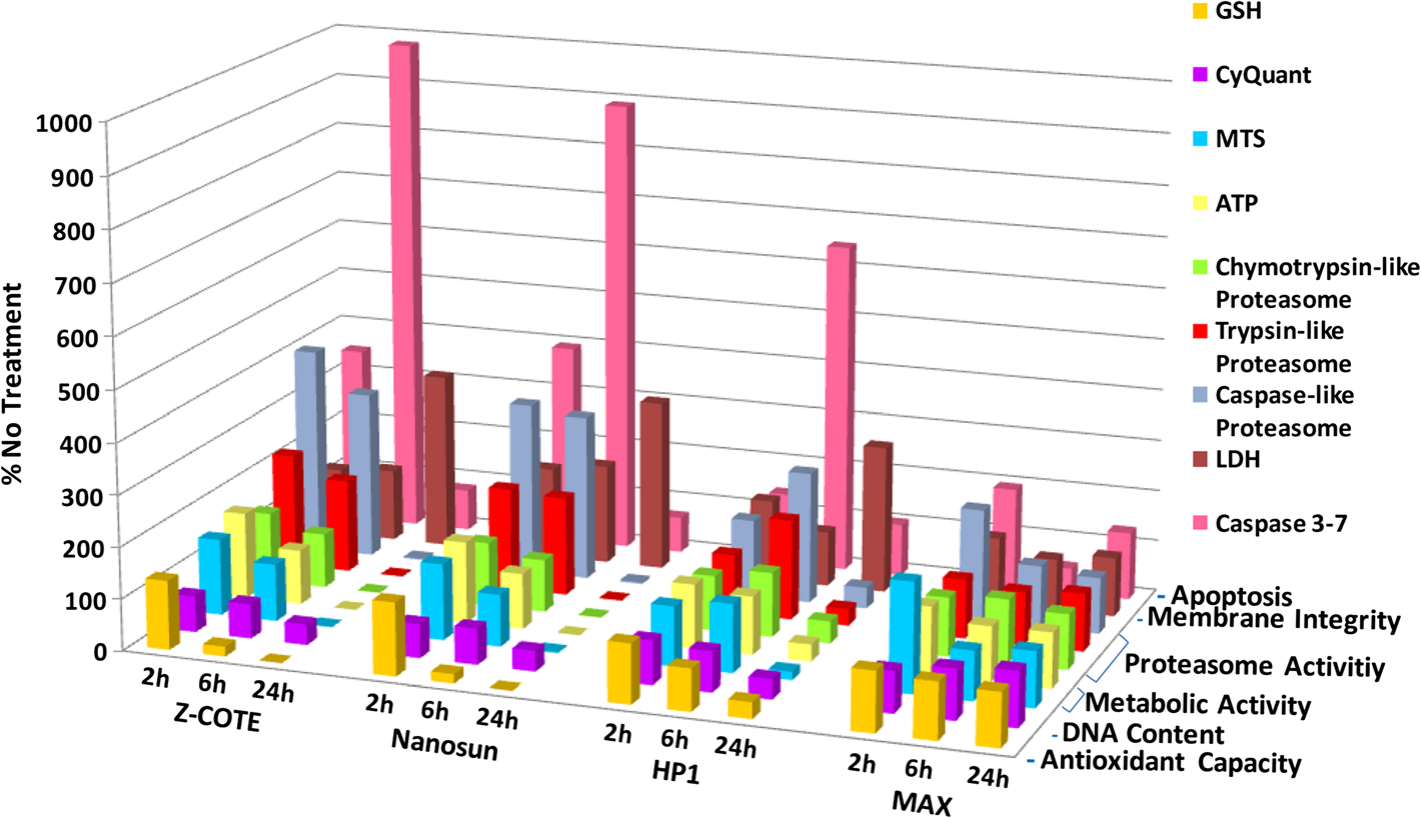
**Changes in cell function in hONS cells treated with ZnO nanoparticles.** For each ZnO treatment at each time-point (2 h, 6 h and 24 h), the responses of hONS cells from donors A, B, C and D, each seeded in three replicate wells, were averaged and expressed as the percentage of time-matched untreated cells set as 100%. Data used to generate this figure, and associated standard errors of means and statistical significance, are supplied separately in Additional file 3: Table S2.

hONS cells from donors A, B, C and D were assessed for viability after exposure to the four ZnO samples for 2, 6 and 24 h, using a range of functional indicators including metabolic activity, antioxidant capacity, proteasome activity, plasma membrane integrity, and apoptosis. Data from the four donors were averaged and the results from all experiments across all time-points are summarised in Figure 7. The data used to generate this figure, together with standard errors of the mean and statistical significance, are supplied separately in Additional file 3: Table S2.

As observed for the cell-signalling assays and transcriptional profiles, treatment with the uncoated particles, Z-COTE and Nanosun, or the coated HP1 induced highly similar responses in terms of both magnitude of change compared to untreated cells and spectrum of cellular functions affected. The most substantial response observed was the elevation of caspase 3–7 activity, indicative of induction of apoptosis by 6 h exposure. The caspase-like and trypsin-like proteasomal activities, associated with targeted protein degradation, were also substantially stimulated by these treatments. By 24 h exposure, cell membrane disruption had risen as measured by an increase in lactate dehydrogenase (LDH) activity in culture medium, and cellular metabolic activity (adenosine triphosphate (ATP) content, methanethiosulfonate (MTS) metabolism, and proteasome activity) had sharply decreased, most likely indicating cell death at this late time-point. In contrast, cells treated with the coated nanoparticle, MAX, showed only a significant increase in MTS metabolism at 2 h, which had subsided to near control levels by 6 h, and these cells were observed to have largely survived treatment after 24 h exposure, as indicated by the control levels of most cell functions at 24 h.

## Discussion

The systemic responses of primary human olfactory neurospheres to four types of commercially-available ZnO nanoparticles are reported here. Two types of ZnO samples had particles of different sizes and were uncoated (Z-COTE and Nanosun), while the other two had particles the same size as Z-COTE but bore different surface coatings (HP1 and MAX), allowing the relative impacts of particle size and applied surface coatings in commercially-produced ZnO samples to be assessed. The responses elicited in human olfactory neurosphere-derived (hONS) cells by all four ZnO samples were generally robust and internally consistent across the comprehensive suite of biological experiments employed, and also across four genetically distinct cell lines in nine cell-stress and viability assays. When first exposed to ZnO nanoparticles, the cells initiated pro-survival responses, generally independent of particle size but slightly dependent on coating, while apoptotic processes dominated after 24 h exposure to three of the ZnO samples - the uncoated Nanosun and Z-COTE, and HP1 coated with triethoxycaprylylsilane. In contrast, cells exposed to MAX, coated with a dimethoxydiphenylsilane/triethoxycaprylylsilane crosspolymer, were still viable at 24 h. Overall, these results suggest that surface coating can be an important factor in mitigating the toxicity of ZnO nanoparticles, while particle size has less impact, at least in the cellular system investigated here.

The minimal toxicity to hONS cells elicited by MAX, compared with HP1, suggests that the magnitude of toxicity reductions conferred by surface coatings may be very sensitive to the chemical composition of the coating molecules; indeed, this has been observed in other cell lines for ZnO nanoparticles bearing a variety of applied coatings [20]. However, other, more subtle characteristics of the coating may also be important. Our comparative analysis of the surface coatings on HP1 and MAX indicates fewer coating molecules per unit surface area on HP1, which could be manifested by HP1 having a thinner coating, a less densely-packed coating, or a patchy coating, and these characteristics could also affect the extent of toxicity mitigation. Furthermore, even if a coating is uniform at the point of manufacture, the strength of interaction between the coating material and the ZnO surface may vary for different coatings (especially if the coating is physically adsorbed) and the coating may partially detach with time and/or in cell culture medium, resulting in a non-uniform coverage of the surface. We have no direct data on the structural characteristics of the coating on HP1 in the cellular system used here. However, consistent with an incomplete surface coverage for HP1 is our observation that the responses in HP1-treated cells clustered with those of cells treated with the uncoated nanoparticles, although delayed by a few hours; in contrast, the cellular responses to MAX were either substantially delayed or minimal. Furthermore, we note that the specific batch of HP1 used here dispersed more readily in cell-culture medium than two other batches of HP1 used by our laboratory, consistent with different extents of surface coverage. We are currently conducting a full physico-chemical analysis of HP1 from the three different batches [36]. Our initial observation of different dispersabilities raises the broader question of the impact of batch-related differences in assessments of nanoparticle toxicity. Given the list of challenges and considerations currently associated with the *in vitro* testing of nanomaterials, taking into account potential batch-to-batch variations appears to be a daunting prospect, but highlights the importance for full nanoparticle characterisation.

Overall, it is tempting to attribute the relative cellular responses to the ZnO samples largely, if not completely, to different concentrations of zinc ions sourced from the dissolution of ZnO particles with varying exposed surface areas. It is feasible that a larger area of exposed particle surface might facilitate a more rapid increase in Zn^2+^ ion concentration compared to a coated or smaller area of exposed surface. Consistent with ZnO nanoparticle literature pointing to zinc ion-mediated toxicity [12,13], a number of the phenotypic outcomes reported here (loss of cellular viability, increase in caspase 3–7 and decrease in cellular glutathione (GSH)) also have been observed as cellular outcomes following *in vitro* treatment of neuronal cells with several types of zinc salt [37]. Furthermore, one of the key factors in cytokine stimulation is the rate of intracellular ion release after nanoparticle uptake by phagocytic cells, which appears to be independent of cytotoxicity [33]; and the increased level of IL-6 at 2 h observed here for the uncoated Nanosun, compared with the uncoated Z-COTE and coated HP1, is consistent with its larger specific surface area and hence a faster release of Zn^2+^ ions than might be expected for Z-COTE and HP1, with the coating on the latter also diminishing its dissolution rate. (In contrast, the cellular responses to the coated MAX are not consistent with zinc ion-mediated cytotoxicity, and the only significant response by the cells to MAX, namely high levels of IL-6 at 2 h and IL-8 at 6 h, may have been induced by the coating itself; this hypothesis has yet to be tested.) However, at odds with a zinc ion-mediated toxicity profile, hONS cells exposed to the uncoated Nanosun and Z-COTE exhibited similar responses, despite a 2.5-fold difference in powdered surface area. Furthermore, a recent report has questioned the extent to which Zn^2+^ ions from zinc salts are actually bio-available in cell culture medium [38]. That report instead showed that ionic zinc swiftly forms a range of insoluble carbonate and phosphate-based nanoparticulate complexes when added to cell culture medium. It is thus possible that zinc salts used as a control for dissolved zinc may form and mimic the effects of nanoparticles themselves, compromising their intended purpose. The range of cytotoxicities associated with different counter ions of zinc salts [37], alongside conflicting reports on zinc salt toxicity [12,13] compared to ZnO nanoparticles [39-41], and the possibility that zinc ions may themselves form a range of nano-complexes in cell culture, together suggest that further consideration of the role of Zn^2+^ in ZnO nanoparticle toxicity experiments is warranted.

A distinct spectrum of key cell-stress signalling pathways was most rapidly activated by the uncoated ZnO nanoparticles, followed by the coated HP1 particles. In contrast, cells treated with MAX had much less substantial cell-signalling responses, did not demonstrate a cellular stress response, and did not lose viability. This pattern was also reflected at the transcriptional level, where similar pathways associated with oxidative stress and cell survival were activated by all treatments, but consistently more so in cells treated with the uncoated nanoparticles or HP1 compared to MAX. Treatments that induced the strongest cell-signalling and transcriptional perturbations also showed the greatest cell function responses, resulting in the most significant losses in viability.

Specifically, in cells exposed to Z-COTE, Nanosun or HP1, we observed an early activation of the MAPK and Akt cell-signalling pathways, as well as the NF-kB pathway. This cell-signalling fingerprint is associated with inflammation, proliferation and anti-apoptotic responses and suggests that, in the first instance, these cells mounted a protective response. The activation of these pro-survival responses had substantially diminished by 6-10 h, concomitant with a decrease in cellular GSH levels, and increased proteasome and caspase 3-7 activities, indicative of oxidative stress, protein degradation and initiation of apoptosis, respectively. Late responses in these cells (24 h) included significantly compromised cell membrane and a decrease in most other homeostatic metabolic activities, consistent with a treatment-related reduction in cell viability. The increase in proteasomal activity in cells treated with either of the uncoated nanoparticles or the coated HP1, together with the induction of a range of molecular chaperone genes, may suggest the induction of an unfolded protein response [42]. Disregulation of protein folding has similarly been suggested elsewhere as a mode of action following treatment of cells with micro or nano-sized ZnO particles [43]; whether this response would be a cause or effect of treatment-related loss of cellular viability remains to be elucidated. Additionally, at 6 h, we found that a number of histone-related genes tended to be up-regulated with increasing treatment toxicity, but down-regulated by treatment with the more benign MAX. Thus, the cytotoxic nanoparticle treatments may have been associated with a suppression of transcriptional activity in addition to an unfolded protein response.

All ZnO nanoparticles induced an early perturbation of Canonical Gene Pathways associated with oxidative stress and cellular stress responses, with stronger perturbations associated with increasing treatment cytotoxicity. At 2 h and 6 h, a number of metallothioneins, molecular chaperonins, zinc finger proteins and solute carrier (SLC) genes were differentially regulated (generally positively) by all treatments, suggesting the induction of mechanisms to modulate intracellular levels of zinc, as well as a generalised cellular stress response. Using immortalised cell cultures treated with ZnO particles, Moos *et al.* (2011) reported a similar transcriptional profile.

Interestingly, given its role as a key tumor-suppressor protein regulating cellular apoptosis in response to cyto or genotoxic insults, we did not observe a statistically significant increase in p53 phosphorylated at Serine 15 (a site closely associated with DNA-damage response [44]), nor did we see an increase in the activation of either of the cell-cycle checkpoint proteins, Chk1 or Chk2. This contrasts with previous reports of p53 protein up-regulation following *in vitro* treatment with ZnO nanoparticles [41,45,46] using different cell lines. However, consistent with the absence of activation of the cell-signalling pathways involving p-p53, pChk1 and pChk2 here, we also observed that a number of transcripts encoding DNA-damage processing proteins were generally down-regulated at 6 h, with the exception of the *POLH* gene, which was up-regulated by all treatments. The *POLH* gene encodes a polymerase that accurately replicates past thymine-thymine dimers (typically associated with UV-induced DNA damage) during translesion synthesis (TLS), but is otherwise a low-fidelity polymerase when copying undamaged DNA [47]. The up-regulation of a key TLS enzyme alongside the down-regulation of enzymes associated with DNA repair, as well as activation of proliferative pathways and lack of cell-cycle checkpoint activation, may suggest that the hONS cells responded to damage in DNA induced by ZnO nanoparticles by preferentially by-passing lesions rather than repairing them.

There is little reported elsewhere in the literature concerning the impact of ZnO nanoparticles on the activation and function of DNA-damage processing mechanisms, such as base excision repair, nucleotide excision repair, or translesion synthesis. The general down-regulation of transcripts associated with DNA-damage processing reported here may suggest that, even though ZnO nanoparticles have been shown elsewhere to induce DNA-damage [20,41,45,48], primary hONS cells did not respond to this genotoxic insult by activating DNA-damage repair mechanisms, or alternatively that the DNA was not damaged in these experiments. Consistent with the former, Hackenberg [49] showed, following repeated exposure to sub-cytotoxic doses of ZnO nanoparticles, that damage to DNA in human nasal mucosa mini-organ cultures was not repaired, and in fact damage increased during a 24 h recovery period, compared to repair observed in methyl methanesulfonate-treated cells. Different surface coatings have been shown previously to protect cell viability by reducing the generation of ROS from ZnO nanoparticles [21], but not necessarily by reducing nanoparticle-mediated genotoxicity [20]. ZnO nanoparticle-mediated genotoxicity, therefore, may not be a major trigger for concomitant cytotoxicity. Whether ZnO nanoparticle-mediated DNA-damage might be associated with direct inhibition of damage-processing proteins by the particles themselves, or whether the generalised stress responses of increasingly unviable cells overwhelms or bypasses such mechanisms, or a combination of the two, would be an interesting avenue to pursue.

Here, we utilised classical suspension to expose cells rather than the novel technique of delivering nanoparticle aerosols to the air-liquid-interface (ALI) of cell cultures e.g. [50]. The two techniques can produce differences in assay parameters, including deposition kinetics, nanoparticle agglomeration and dissolution, and the influence of cell-culture medium on the surface characteristics of the nanoparticles [51], which can influence biological end-points. Literature comparing the effects of nanoparticles delivered via the two techniques indicate general agreement, but also some differences, in cellular responses over time, including cell membrane integrity, gene expression of pro-inflammatory and oxidative stress markers, and cell viability [50,51]. It was further reported that while toxicity assessments at the ALI are likely to produce the more relevant biological responses, the influence of gas-derived effects can mask those of the nanoparticles [51] and that, for the moment, classical suspension exposures remain a valuable complementary technique when assessing the toxicity of nanoparticles to cells relevant for inhalation exposure.

The actual nanoparticle dose to cells in the olfactory bulb will depend in part on the numbers of nanoparticles inhaled through the nose, and their state of agglomeration. Work by Guilherme and Kimbell [52] showed good agreement between experimentally derived calculations and computer modelling predictions for particle sizes up to 100 nm, suggesting that approximately 20% of nanoparticles deposited in the olfactory region will translocate to the olfactory bulb [23]. While we determined sizes of the individual ZnO nanoparticles and time-dependent changes in sizes of agglomerates forming in cell-culture medium, we did not characterise the particle sizes in aerosol form. Thus, it is difficult to estimate what the nasally-inhaled dose of ZnO particles would be, with subsequent translocation to the olfactory mucosa, that would produce the dose used here for the suspension assays.

## Conclusions

In conclusion, we have reported a comprehensive overview of the response of primary human multi-potent cells to uncoated and coated ZnO nanoparticles in an important *in vitro* model with respect to nasal-inhalation exposure. Our findings support two major conclusions. First, we find that surface coatings on ZnO nanoparticles may delay or substantially mitigate the onset of cellular responses depending on the coating composition and other coating characteristics. Second, whilst ZnO nanoparticle toxicity can be mediated by a range of cellular stress, inflammation and apoptotic responses, mechanisms to process damaged DNA do not appear to be activated, and may be down-regulated. Given the results from our *in vitro* model, we suggest that inhaled, uncoated ZnO nanoparticles may adversely impact local multipotent cell populations in the olfactory mucosa *in vivo*. Thus, depending on the volumes handled, personal protection or engineering controls are recommended for workplaces where aerosols of uncoated ZnO nanoparticles are generated. ZnO nanoparticle toxicity, however, can be largely mitigated by the addition of stable surface coatings.

## Methods

### ZnO nanoparticles

Two types of uncoated ZnO nanoparticles were used in this study: Z-COTE (BASF, batch# EHDA3001) and Nanosun P99/30 (Micronisers, batch# 4051). Two types of coated ZnO nanoparticles were also used: Z-COTE HP1 (BASF, batch# CNHE0602) coated with triethoxycaprylylsilane; and Z-COTE MAX (BASF, batch# FCHE1301) coated with a dimethoxydiphenylsilane/triethoxycaprylylsilane crosspolymer.

### Physicochemical characterisation of nanoparticles

#### Particle size, size distribution, and shape

Sizes and shapes of the ZnO particles were characterised by transmission electron microscopy (TEM) using a Tecnai 12 TEM (FEI, Eindhoven, Netherlands) operating at 120 kV under a variety of magnifications. Prior to measurement, TEM calibration was confirmed by the accurate size determination of 30 nm gold nanoparticle standards supplied by the National Institute of Standards and Technology, USA (RM8012) [53]. Dispersions of nanoparticle powders were prepared by adding small amounts (~1 mg) to 30 μL ethanol and bath sonicating gently for 5 min to form a milky suspension. Samples were prepared for TEM imaging by applying a 4 μL aliquot of suspension to 400-mesh carbon-coated grids freshly glow-discharged for 15 sec in nitrogen. The sample was allowed to settle for approximately 1 min after which excess sample was whicked off with filter paper. Images were recorded using a MegaView III CCD camera (Olympus) and sizes were determined using AnalySis software (Olympus) and Image J software (NIH) calibrated via the embedded scale bar. Only the particle diameter was measured for spherical particles (Nanosun), whereas both width and length were measured for variously shaped particles (Z-COTE, HP1 and MAX). 90–300 measurements were taken for each dimension.

### Specific surface areas of particles

The Brunauer-Emmett-Teller (BET) equation was used to calculate specific surface areas of the ZnO powders from nitrogen adsorption isotherms measured at 77 K using a Micromeritics ASAP 2010 volumetric adsorption analyser [54]. The BET calculation was performed from the adsorption isotherm at relative pressure (P/Po) ranging from 0.05 to 0.2. Before measurement, samples were out-gassed at 120°C for over 24 h. A second set of measurements was performed by a different operator in a different laboratory using a different Micromeritics instrument, to check original measurements.

### Free radical generation by particles

Levels of peroxynitrile and superoxide radicals generated by the ZnO particles were determined in triplicate by electron paramagnetic resonance (EPR) using TEMPONE-H (Enzo Life Sciences) as a spin trap. Test samples (0.01 mg/mL) were prepared in sterile saline. TEMPONE-H (1 μL of 100 mM stock solution) was added to 99 μL of the test sample to obtain a final concentration of 1 mM TEMPONE-H. The samples were incubated at 37°C for 60 min either in complete darkness or in sunlight filtered through a window, after which the levels of oxidised TEMPONE-H were quantified by EPR. A negative control was prepared using saline only.

### Analysis of surface coatings in HP1 and MAX

Elemental compositions of the coated HP1 and MAX were determined using 0.15 g of sample dissolved in a 1:1 HNO_3_: H_2_O_2_ mixture with heating for 30 min. The solution was diluted to 100 mL, internal standard Sc was added, and the resultant solution was analysed by Inductively Coupled Plasma-Atomic Emission Spectroscopy (Varian 730 Axial ICP-AES).

Thermal properties of the coated HP1 and MAX were analysed with a thermogravimetric analyser (Mettler Toledo TGA SDTA851 with a Mettler TSO 801 RO sample robot (Mettler Toledo, Melbourne, Australia)). Approximately 60 mg (accurately weighed) of powdered samples in ceramic crucibles were heated in a carrier gas of air at the rate of 10°C/min from room temperature to a maximum of 1100°C, and weight loss as a function of temperature was recorded.

### Endotoxin testing

For measurement of endotoxin levels, 1 mg/mL each sample was vortexed for 1 min in limulus amebocyte lysate (LAL) endotoxin-free water and incubated for 1 h at 37°C. Samples were then centrifuged and endotoxin levels in the supernatant were determined in triplicate using the QLC-1000 Chromogenic LAL kit (Lonza) following manufacturer’s instructions. A previous trial had shown that centrifugation did not artificially lower endotoxin levels in the supernatant (data not shown). An aliquot of each supernatant was also spiked with a known amount of endotoxin and measured alongside unspiked samples to check if the nanoparticles inhibited the assay. The spiked sample for Z-COTE indicated minor assay inhibition, but retesting using diluted samples showed that endotoxin levels remained below the assay detection limit (<10 pg/mL). Nanosun, HP1 and MAX did not inhibit the assay.

### Characterisation of nanoparticles dispersed in cell culture medium

Nanoparticle suspensions (25 μg/mL) were prepared in DMEM/F12 (1:1), and incubated (37°C, 10% CO_2_) for 0, 2, 6 and 24 h. Within 30 min of removal from the incubator, hydrodynamic particle size and surface charge were determined by dynamic light scattering (DLS) and measurement of zeta potential, respectively (Zetasizer Nano series, Malvern Instruments). All measurements were performed at 37°C to simulate the temperature at which cells were incubated. Hydrodynamic particle size in DMEM/F12 medium at 0 h was also assessed by differential centrifugal sedimentation (DCS) (CPS DC24000 UHR Disc Centrifuge, LPS Instruments, Inc.). The pH of cell-culture medium containing nanoparticles was measured at 0, 2, 6 and 24 h using a calibrated pH meter (Waterproof pHTestr2, Oakton Instruments).

### Cell cultures

Neurosphere-derived cells generated from four individual olfactory mucosa biopsies (from human donors A, B, C and D) were propagated as adherent monolayers in DMEM/F12 (1:1, Gibco) containing 10% FBS, as described elsewhere [31]. The biopsies were obtained after informed consent, and the procedure was approved by the Griffith University Human Ethics Committee and according to guidelines of the National Health and Medical Research Council of Australia. For all experiments described here, cells were used between passages 4-15 from neurospheres that had been generated once from primary cultures between passages 5-8, depending on the donor cell line. All incubations were performed at 37°C, 10% CO_2_.

### Preparation of nanoparticle test suspensions and controls

A stock suspension (1 mg/mL) of each ZnO product was prepared by adding cell-culture medium to pre-weighed powder in a clean, sterile 50 mL Falcon tube. Tubes were briefly vortexed and then bath sonicated for 15 min to disperse the nanoparticles. The stock suspension was diluted with medium to the appropriate test concentrations and then sonicated for a further 15 min before being added to cell cultures.

### Selection of nanoparticle concentration for detailed investigations of biological responses

Cells from donor A were seeded (2500 cells/well) into two 96-well plates (one clear, one opaque black) and incubated overnight. Medium was removed and replaced in duplicate wells with medium containing 10, 20, 30, 50 or 80 μg/mL nanoparticles, or medium containing no nanoparticles, and incubated, providing duplicate measurements for each assay and each treatment. At 22 h, MTS reagent (20 μL; CellTiter Aqueous MTS assay, Promega) was added to each well of the clear plate as a measure of cell viability via mitochondrial activity, and cells were incubated for a further 2 h; and, at 23 h, CyQUANT reagent (50 μL; Invitrogen) was added to each well of the black plate to determine cell number via DNA content, and cells were incubated for a further 1 h, following the manufacturer’s instructions. After a total of 24 h incubation, plate absorbance/fluorescence was recorded according to the manufacturer’s instructions using a Synergy II plate reader (BioTek).

Data from cells in each well were corrected for background, using signal values from wells containing medium alone or from matched concentrations of nanoparticles incubated and assayed in the absence of cells.

### Cytokine assays

Cells from donor A were seeded (120,000 cells/well) into 6-well plates and incubated overnight. Medium was removed, and replaced with medium containing either 25 μg/mL nanoparticles, or no nanoparticles, and then incubated for 2 h or 6 h. Three replicate wells were used for each treatment. After incubation, conditioned medium was taken from the wells and frozen at −80°C.

Cytokine protein levels were determined using a Th1/Th2 11-plex bead array kit (Bender MedSystems). Beads were analysed via flow cytometry on a Canto II digital flow cytometer (BD Biosciences). Individual bead populations were discriminated by size and fluorescence at 620/633 nm (excitation/emission) and the amount of cytokine binding was assessed by phycoerythrin (PE) fluorescence (488/575 nm). Cytokine signal was measured as mean PE fluorescent intensity in each population and compared to known standards. Curve fitting and calculation of cytokine concentrations in the media were performed using GraphPad Prism 5.01 (GraphPad Software, USA) and assessed for statistical significance by one-way ANOVA with Bonferroni’s multiple comparison test, set at *p*<0.05.

### Cell signalling assays

Cells from donor A were seeded (55,000 cells/well) into 12-well plates and cultured overnight. Medium was removed and replaced with medium containing either 25 μg/mL nanoparticles, or no nanoparticles, for 2, 4, 6, 8, and 10 h. Three replicate wells were used for each treatment. At the appropriate time-points, the medium was removed from the wells and the cells were lysed with 500 μL 1X AlphaScreen SureFire Lysis buffer. The lysates were frozen at -80°C until further analysis.

The cell lysates were analyzed for phosphorylation (p) of several intercellular signalling proteins using the AlphaScreen SureFire platform (PerkinElmer) following manufacturer’s instructions. The specific proteins examined were Akt (p-Ser473), BAD (p-Ser112), MEK1 (p-Thr217/Tyr221), ERK (p-Thr202/Tyr204), p38 MAPK (p-Thr185/Tyr187), c-JUN (p-Ser63), JNK (p-Thr185/Tyr187), NF-kB p65 (p-Ser536), I-kΒα (p-Ser32/36), p53 (p-Ser15), Chk1 (p-Ser), and Chk2 (p-Ser45). Levels of total (t) ERK, Akt and I-kΒα were also measured as controls to ensure signalling protein levels were not generally changing over the time course of the experiments.

Briefly, the lysates were thawed, and portions of lysate (4 μL) were transferred to wells in assay plates (384-Proxiplates, PerkinElmer) and analyzed in parallel for the formation of specific phosphorylated epitopes on several key intracellular signalling proteins, as previously described in detail [55]. The signals in the wells were determined using an Envision multilabel plate reader (PerkinElmer), using standard Alpha settings.

Time-course responses were analyzed using GraphPad Prism 5.01 (GraphPad Software, USA). Differences over time between treatments versus no treatment were assessed for statistical significance by 2-way ANOVA with Dunnett’s multiple comparisons test using GraphPad Prism 5.01 (GraphPad Software, USA), where statistical significance was set at *p*<0.05.

### Gene expression assays using microarrays

Cells from donor A were treated as described above for the cytokine assays. Four replicate wells for each treatment were used. After aspiration of medium, cells were lysed in the well, and RNA was isolated using a NucleoSpin® RNA II kit (Macherey-Nagel, Scientifix) following manufacturer’s instructions. RNA concentration was determined using a NanoDrop DN-1000 spectrophotometer (Biolab), and RNA integrity (i.e. no, or limited, degradation) was confirmed (RNA 6000 Nanochip™, 2100 Bioanalyzer; Agilent Technologies). Four samples per treatment (a total of 56 samples) were prepared for microarray analysis using the Affymetrix GeneChip Human Gene 1.0 ST Array Combo kit (Millennium Science) containing arrays and reagents, following manufacturer’s instructions. Microchips were prepared for hybridisation and scanning using an Affymetrix GeneChip® Hybridisation, Wash and Stain Kit (Millennium Science), following manufacturer’s instructions. After hybridisation (17 h, 45°C, 60 rpm in an Affymetrix 640 GeneChip® Hybridization Oven), microchips were washed using an Affymetrix GeneChip® Fluidics Station 450 and scanned using an Affymetrix 7G GeneChip® Scanner. All microchips passed the associated quality control procedures recommended by Affymetrix.

The microchips were processed in eight batches of seven by the same operator, and following the same procedure. Each batch was processed on a different day, and contained one microchip from each of the four time-matched treatments (HP1, Z-COTE, Nanosun and MAX), controls, plus two other treatments which were not subjects of the present study. The gene expression data were normalized by applying the robust multi-array average (RMA) algorithm using Matlab 7.7.0 (The Mathworks Inc., Natick, MA, USA) on the entire data set from all 56 microchips (28 from the 2 h treatments and 28 from the 6 h treatments), treated as a whole.

Final gene datasets were analyzed through the use of Ingenuity Pathways Analysis (Ingenuity® System, http://www.ingenuity.com), taking into account whether the genes were under-expressed or over-expressed. Overlapping responses between treatments were identified on the basis of shared over-expressed and under-expressed genes, as well as shared activation of a gene network.

Gene expression datasets, like other high throughput datasets, suffer from what are known as batch effects [56,57]. In essence, batch effects are structured patterns of distortion caused by the interaction of various sources of noise associated with the laboratory processing of high throughput arrays. This distortion is unique to each batch, with significant variation from batch to batch. Therefore, even when the processing batches are balanced across treatments as is the case in this study, batch effects masquerade as within-treatment variance reducing significantly the power of statistical comparison tests. There are techniques based on model fitting to remove batch effects, but these always have the risk of over-correction, resulting in the removal of genuine biological variance. Removing genuine variance has the undesirable effect of leading to false positives – genes that appear to be differentially-expressed when in fact they are not. In this study we have employed a technique [58] that quantifies the risk of over-correction, and then enables the maximal removal of batch noise with the constraint that this risk is controlled at a probability level set by the experimenter (in our case we set this probability to be .05).

For each treatment, and for each probe-set included in the microchips, the four treatment scores were compared against four control scores via t-tests. Given that the probe-sets on the arrays number in the thousands, the next challenge was to implement a multiplicity correction process that strikes a sensible balance between Type 1 and Type 2 errors – that is between controlling false positives and false negatives. Traditional correction techniques (such as Bonferroni) do reject false positives when applied to microarray data, but this comes at the expense of a typically high number of false negatives. We employed a hybrid method [59] which took its inspiration from biologists’ “fold-change” test. Based on empirical distributions from the probe-set scores, we derived expected rates of false discovery as a function of the absolute log_2_ difference between the treatment mean and the control mean. The gene associated with the probe-set was deemed differentially-expressed for a given treatment if there was an absolute log_2_ difference of ‘x’ between the treatment mean and the control mean (i.e. that one mean was 2^x^ times the other), where ‘x’ corresponded to a false discovery rate of 0.1, with the further condition that this difference was statistically significant as tested by a 2-way t-test set at *p*=0.05. If this difference was positive, the gene was deemed over-expressed, and if negative, under-expressed. In this way, we were able to establish lists of differentially-expressed genes for individual treatments, which could be compared to one another. In the traditional fold-change test, ‘x’ is typically set to 1.0 for all treatments, but this is problematic in comparing different treatments because, depending on the probe-set distributions, a log_2_ difference of 1.0 between treatment and control will correspond to different false discovery rates across different treatments.

### Cell stress and viability assays

Cells from donors A, B, C and D were seeded (2500 cells/well) into 96-well plates (clear, or opaque black or white, depending on assay requirement) and incubated overnight. Medium was removed and replaced with medium containing either 25 μg/mL nanoparticles, or no nanoparticles, and incubated for 2, 6 or 24 h. Three replicate wells for each treatment were used for each assay and for each cell line. Towards the end of each incubation period, plates and reagents were equilibrated to room temperature and cell function assay reagents were added to each plate to test for NAD(P)H-dependent dehydrogenase activity (CellTiter Aqueous MTS assay, Promega), ATP levels (ATPlite assay, Perkin Elmer), lactate dehydrogenase (LDH) activity (CytoTox-ONE homogenous membrane integrity assay, Promega), reduced and total glutathione (GSH) content (GSH-Glo glutathione assay, Promega), chymotrypsin-like, trypsin-like and caspase-like proteasome activities (Proteasome-Glo cell-based assays, Promega), and caspase 3-7 activity (Apo-ONE homogenous caspase-3/7 assay, Promega) according to manufacturer-supplied protocols with only minor modifications as described previously [60] where well absorbance/luminescence/fluorescence was recorded using a Synergy II plate reader (BioTek).

Signals from wells containing cells were corrected for background by subtracting the signal from wells containing medium alone, and signals from wells with cells and nanoparticles were corrected by subtracting the signal from 25 μg/mL nanoparticles in medium in the absence of cells. Mean background-corrected results for each donor from triplicate wells were then averaged across the four donors to give a final value for each treatment. To correct for a possible decrease in signal due to loss of live cells rather than treatment-mediated effects on cell function, data for cell function assays were normalised to treatment-matched DNA content in live cells (CyQUANT-NF, Invitrogen).

Data from each treatment were assessed for statistical significance by 2-way ANOVA with Bonferroni’s multiple comparisons test set at *p*<0.05, using GraphPad Prism 5.01 (GraphPad Software, USA).

## Abbreviations

AIL: Air-liquid-interface; ATP: Adenosine-triphosphate; BD: Below detection; BET: Brunauer-Emmett-Teller; C: Celsius; chk: Checkpoint; CO2: Carbon dioxide; DCS: Differential centrifugation sedimentation; DLS: Dynamic light scattering; DMEM: Dulbecco’s modified eagle medium; DNA: Deoxyribonucleic acid; EPR: Electron paramagnetic resonance; ERK: Extracellular signal-related kinase; FBS: Fetal bovine serum; g: Gram; GSH: Glutathione; h: Hour; hONS: Human olfactory neurosphere-derived cells; ICP-AES: Inductively coupled plasma atomic emission spectroscopy; IFN: Interferon; IkB: I-kappaB; IL: Interleukin; IPA: Ingenuity pathway analysis; JNK: c-Jun N-terminal kinase; K: Kelvin; LAL: Limulus amebocyte lysate; LDH: Lactate dehydrogenase; m: Meter; MAPK: Mitogen-activated protein kinase; MEK: Mitogen-activated protein kinase kinase; mg: Milligram; min: Minutes; mL: Millilitre; mM: Millimolar; MTS: Methanethiosulfonate; N/A: Not applicable; NIST: National Institute of Standards and Technology; nm: Nanometer; NFkB: Nuclear factor kappaB; NS: Not significant; p: Phosphorylated; PE: Phycoerythrin; pg: Picogram; RMA: Robust multi-array average; RNA: Ribonucleic acid; ROS: Reactive oxygen species; rpm: Revolutions per minute; sec: Seconds; SLC: Solute carrier; TEM: Transmission electron microscopy; TGA: Thermogravimetric analysis; TLS: Translesion synthesis; TNF: Tumour neurosis factor; Zn: Zinc; ZnO: Zinc oxide; μg: Microgram.

## Competing interests

The authors declared that they have no competing interests.

## Authors’ contributions

MJO drafted the manuscript and contributed to experimental work and data analysis. ALC, RIWO, and BF contributed to experimental work, data analysis and manuscript preparation. YO contributed to data analysis and manuscript preparation. MJM, AM-S and SAW oversaw all experimental work and contributed to manuscript preparation. All authors read and approved the final manuscript.

## Supplementary Material

Additional file 1: Figure S1 hONS cell viability across a range of ZnO nanoparticle concentrations (10–80 μg/mL). For each ZnO treatment at 24 h, the responses of hONS cells from donor A, in two replicate wells, were averaged and expressed as the percentage of time-matched untreated cells set as 100%. Cell viability was assessed using A. the MTS assay, and B. the CyQuant Assay. Click here for file

Additional file 2: Table S1 Phosphorylation of proteins in key cell-signalling pathways in hONS cells exposed to ZnO nanoparticles. Click here for file

Additional file 3: Table S2 Functional changes in hONS cells exposed to ZnO nanoparticles. Click here for file
