# Supplementary figures and images for: Surface coatings of ZnO nanoparticles mitigate differentially a host of transcriptional, protein and signalling responses in primary human olfactory cells
Source: Part Fibre Toxicol. 2013 Oct 21;10:54. doi: 10.1186/1743-8977-10-54 (PMC4016547; doi:10.1186/1743-8977-10-54)

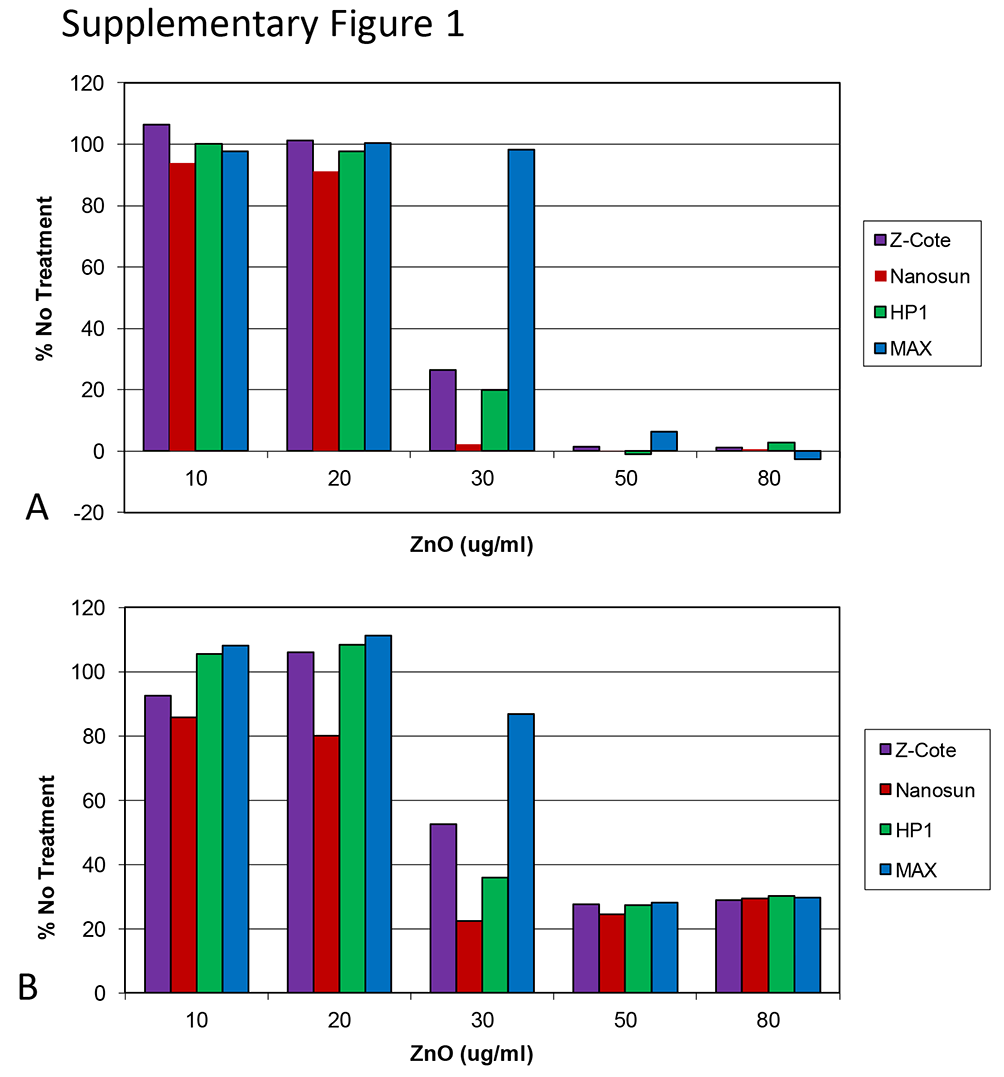

Supplement: Additional file 1: Figure S1 — hONS cell viability across a range of ZnO nanoparticle concentrations (10–80 μg/mL). For each ZnO treatment at 24 h, the responses of hONS cells from donor A, in two replicate wells, were averaged and expressed as the percentage of time-matched untreated cells set as 100%. Cell viability was assessed using A. the MTS assay, and B. the CyQuant Assay. [file 1743-8977-10-54-S1.tiff]
