# Supplementary material for: Surface coatings of ZnO nanoparticles mitigate differentially a host of transcriptional, protein and signalling responses in primary human olfactory cells
Source: Part Fibre Toxicol. 2013 Oct 21;10:54. doi: 10.1186/1743-8977-10-54 (PMC4016547; doi:10.1186/1743-8977-10-54)
Supplement: Additional file 2: Table S1 — Phosphorylation of proteins in key cell-signalling pathways in hONS cells exposed to ZnO nanoparticles. [file 1743-8977-10-54-S2.docx]

%NT: Percent no treatment cells; SEM: Standard error of the mean. Red cells indicate concentrations were significantly less than in untreated cells. Green cells indicate concentrations were significantly greater than in untreated cells.
